# Polyketides and alkaloids from the fungus *Aspergillus Fumigatus* YB4-17 and *ent*-Fumiquinazoline J induce apoptosis, paraptosis in human hepatoma HepG2 cells

**DOI:** 10.3389/fphar.2024.1487977

**Published:** 2024-12-04

**Authors:** Huannan Wang, Lixiang Sun, Xueyang Ma, Shihao Jin, Yidan Xi, Chunmei Sai, Maocai Yan, Zhongbin Cheng, Zhen Zhang

**Affiliations:** ^1^ School of Pharmacy, Jining Medical University, Rizhao, Shandong, China; ^2^ School of Pharmacy, Binzhou Medical University, Yantai, Shandong, China; ^3^ Key Laboratory of Tropical Biological Resources of Ministry of Education, School of Pharmaceutical Sciences, Hainan University, Haikou, China

**Keywords:** *Aspergillus fumigatus*, polyketides, alkaloids, apoptosis, paraptosis, HCC HepG2 cells

## Abstract

Hepatocellular carcinoma (HCC) is one of the most common malignancies. The currently available clinical drugs for HCC frequently cause serious side effects and the treatment outcomes are unsatisfactory. It is urgent to develop effective drugs with high selectivity and low adverse effects for HCC. Metabolites produced by microorganisms have shown great potential in the development of therapeutic agents for HCC. In our study, the EtOAc extract of the strain *Aspergillus fumigatus* YB4-17 exhibited significant cytotoxicity towards the HCC HepG2 cells at 10 μg/mL. Various column chromatographic separations of the extract afforded seven polyketides (**1**–**7**), including a new diphenyl ether derivative (**1**), along with fourteen known alkaloids (**8**–**21**). The structure elucidation was conducted via NMR spectroscopic data and MS data analysis. The absolute configuration of compound **11** was confirmed by comparing experimental and calculated electronic circular dichroism spectrum for the first time. The biological evaluation of these metabolites revealed that compound **11** selectively inhibited the proliferation of HCC HepG2 cells with negligible toxicity to normal cells. Mechanism study indicated that compound **11** induced apoptosis and paraptosis in HepG2 cells, providing a novel therapeutic perspective for the treatment of hepatocellular carcinoma.

## 1 Introduction

Hepatocellular carcinoma (HCC) is one of the most common malignancies. Globally, HCC ranks fifth in incidence among all cancers, while its mortality rate is the second highest (Sung, et al., 2021; Ferlay, et al., 2015). In China, the incidence and mortality rates of HCC are on the rise, the statistics prove that it is one of the leading causes of cancer-related deaths (Han, et al., 2024). Despite recent advancements in therapeutic techniques for HCC, the 5-year survival rate for patients remains discouragingly low at 14.1% (Fitzmaurice, et al., 2019). Currently, clinical drugs for HCC mainly include chemotherapeutic agents, targeted therapy drugs, and immunotherapeutic agents. However, these drugs frequently cause serious side effects and the treatment outcomes are unsatisfactory (Brown et al., 2023). The aforementioned information indicates it is urgent to develop effective drugs with high selectivity and low adverse effects for HCC.

Natural products have garnered significant attention due to their noteworthily activity and low toxicity profiles (Saini, et al., 2023; Guo, et al., 2024; Dalisay, et al., 2024; Rai, et al., 2024). In particular, microorganism metabolites show great potential in the development of therapeutic agents for HCC (Yuan, et al., 2020; Deshmukh, et al., 2017; Blessie, et al., 2020; Dai, et al., 2021; Ran, et al., 2020). For instance, bleomycin, originally isolated from *Streptomyces*, has been widely utilized in the clinical treatment of HCC (Wang, et al., 2018; Fu, et al., 2021; Kacała, et al., 2024). Marine fungi constitute a significant reservoir of lead compounds, especially the genus *Aspergillus*, which thrives in marine ecosystems. Previous studies have indicated that the secondary metabolites of marine *Aspergillus* fungi are distinguished by their remarkable pharmacological properties, including anticancer, antimicrobial, anti-inflammatory, antifungal, antiviral, and enzyme-inhibitory activities (Li, et al., 2023a; Shi, et al., 2024; Sun, et al., 2022).

**FIGURE 1:**
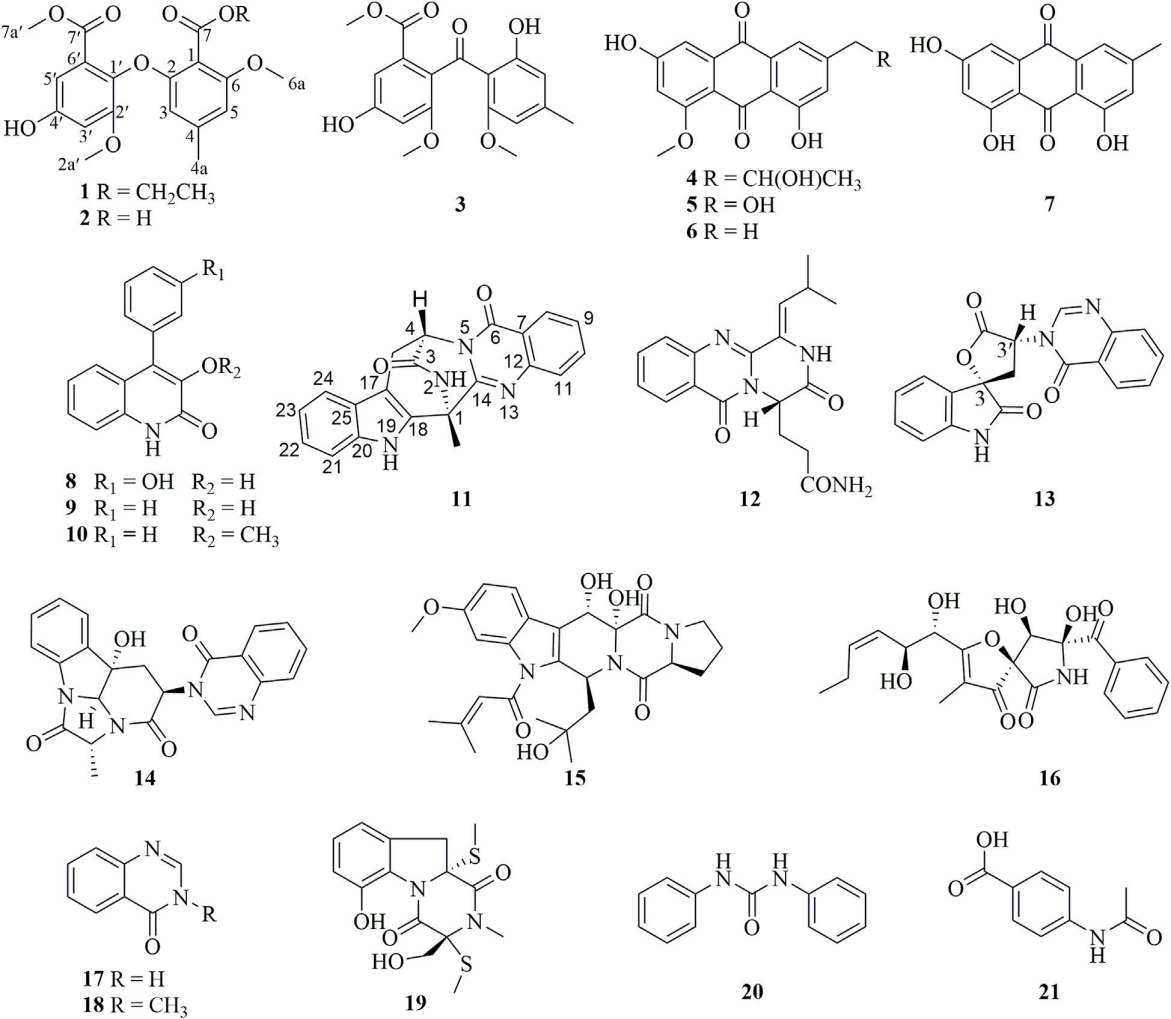
Polyketides (**1**–**7**) and alkaloids (**8**–**21**) from the fungus *Aspergillus fumigatus* YB4-17.

In our efforts to search for bioactive molecules from marine *Aspergillus* strains (Zhang, et al., 2024; Li, et al., 2023b; Cheng, et al., 2019; Cheng, et al., 2016), the EtOAc extract of the marine oyster-derived *Aspergillus fumigatus* YB4-17 exhibited significant cytotoxicity, with a 72% inhibition rate toward the HCC HepG2 cells at the concentration of 10 μg/mL. Various column chromatographic separations of the EtOAc extract afforded seven polyketides (**1**–**7**) and fourteen alkaloids (**8**–**21**) (Figure 1), including two diphenylether derivatives (**1**–**2**), four anthraquinone derivatives (**3**–**7**), six quinazoline-containing alkaloids (**11**–**14**, **17**, and **18**), and two indole alkaloids (**15** and **19**). Compound **1** was a new diphenyl ether derivative. The cytotoxic effect of all compounds was evaluated against a panel of human cancer cell lines, including HepG2, MDA-MB-231, and SW620, as well as a normal human lung epithelial cell line, Beas-2B. Compound **11** demonstrated remarkable selectivity in inhibiting the proliferation of HCC HepG2 cells, with negligible toxicity to normal cells. This paper presents the details of the isolation, structure elucidation, and bioassay results.

## 2 Results

### 2.1 Structure elucidation

Circinophoric acid ethyl ester (**1**) was obtained as a white powder. The chemical formula was assigned as C_20_H_22_O_8_ by HRESIMS ([M + Na]^+^ *m/z* 413.1235, calcd for C_20_H_22_O_8_Na^+^, *m/z* 413.1212) and ^13^C NMR spectroscopy (Supplementary Figure S2), requiring ten degrees of unsaturation. Summation of the integrals from the ^1^H NMR spectrum (Supplementary Figure S2) indicated the presence of a total of 21 protons, indicating the existence of one hydroxy group. Further examination of the ^1^H NMR spectrum indicated the presence of five methyls including a methyl triplet (δ_H_ 1.23, *J* = 7.1 Hz), three methoxys (δ_H_ 3.76, 3.68, 3.61), and an aromatic methyl (δ_H_ 2.15). The observation of two meta-coupled doublets (δ_H_ 6.76, 6.73) and two broad singlets (δ_H_ 6.51, 5.79) in the aromatic region revealed the presence of at least two benzene rings. The quartet integrated for two protons at 4.21 was coupled to the methyl triplet at 1.23, indicating the presence of an ethoxy group. The ^13^C NMR spectrum displayed a total of 20 carbon resonances (Table 1), which could be assigned with the aid of the ^1^H NMR and HSQC spectra to three methoxy carbons (δ_C_ 56.1, 55.9, 52.1), two methyl carbons (δ_C_ 21.6, 14.1), an oxygenated methylene carbon (δ_C_ 60.4), twelve aromatic carbons for two benzene rings (δ_C_ 104.8, 105.3, 105.8, 107.4, 110.0, 125.9, 133.4, 140.6, 153.5, 155.3, 155.9, 156.6), and two ester carbonyl carbons (δ_C_ 165.4, 165.3). The above mentioned data indicated the presence of either two 1,2,3,5-tetrasubstituted benzene moieties or one 1,2,3,5-tetrasubstituted and 1,2,4,5-tetrasubstituted benzene ring. These structural features were very similar to that of the coisolated analogue circinophoric acid (**2**) (Buttachon, et al., 2016), whose structure was unambiguously determined by single-crystal X-ray diffraction method. The only difference between **1** and **2** was found to be the presence of one more ethoxy group (δ_H_ 4.21, 1.23; δ_C_ 60.4, 14.1).

**TABLE 1:** ^1^H NMR and^13^C NMR Data of **1–2** (*δ* in ppm) in DMSO-*d*_4_.

No., type	1		2	
	δ_H,_ mult. (*J* in Hz) ^a^	δ_C_ ^b^	δ_H,_ mult. (*J* in Hz) ^a^	δ_C_ ^b^
1, C		110.0		111.3
2, C		155.9		155.6
3, CH	6.51, br s	105.3	6.49, br s	105.2
4, C		140.6		139.8
5, CH	5.79, br s	105.8	5.76, s	105.8
6, C		156.7		156.4
7, C		165.4		166.5
8, CH_3_	2.15, s	21.6	2.14, s	21.6
1′, C		133.4		133.6
2′, C		153.5		153.5
3′, CH	6.76, d (2.8)	104.8	6.76, d (2.7)	104.8
4′, C		155.3		155.2
5′, CH	6.73, d (2.8)	107.4	6.74, d (2.7)	107.4
6′, C		125.9		125.9
7′, C		165.3		165.5
6-OCH_3_	3.76, s	55.9	3.76, s	55.7
7-CH_2_CH_3_	4.21, q (7.1)	60.4		
7-CH_2_CH_3_	1.23, t (7.1)	14.0		
2′-OCH_3_	3.68, s	56.0	3.69, s	56.1
7′-OCH_3_	3.61, s	52.1	3.61, s	52.1

^a^ at 400 MHz.

^b^ at 100 MHz.

**FIGURE 2:**
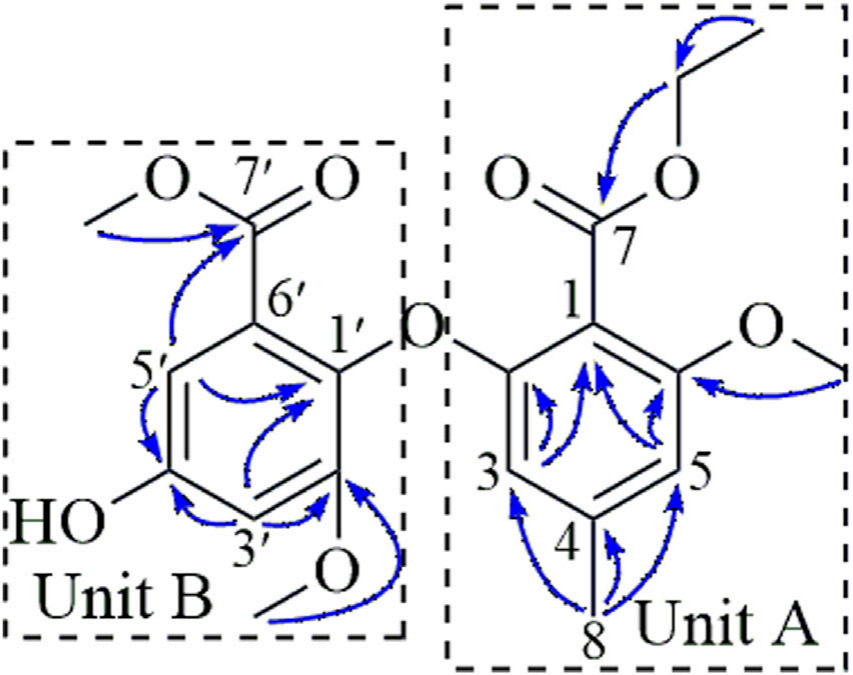
Key HMBC (blue curved arrows) correlations of **1**.

The structure was determined to be a 7-ethyl ester derivative of compound **2** by detailed analysis of the 2D NMR data (Figure 2), compound **1** was a biphenyl ether derivative containing two benzoate units (A and B). In unit A, the HMBC correlations from H_3_-8 (δ_H_ 2.15) to C-3 (δ_C_ 105.3), C-4 (δ_C_ 140.6), C-5 (δ_C_ 105.8) located an aromatic methyl group at C-4 (δ_C_ 140.6). Additional HMBC correlations from H-3 (δ_H_ 6.51) to C-1 (δ_C_ 110.0), C-2 (δ_C_ 155.9) and H-5 (δ_H_ 6.51) to C-1, C-6 (δ_C_ 156.7) positioned two O-substituents at C-2 and C-6, respectively, as well as a C-substituent at C-1. The HMBC correlation from the methoxy protons at 3.76 to C-6 positioned a methoxy group at C-6. The ethoxy group was linked to the carbonyl carbon C-7 (δ_C_ 165.4) via HMBC correlation from the methylene protons (δ_H_ 4.21) to C-7 (δC 165.4) to form an ethyl ester moiety connected to C-1. In unit B, the HMBC correlations from H-5′ (δ_H_ 6.73) to C-1′ (δ_C_ 133.4), C-4′ (δ_C_ 155.3) and from H-3′ (δ_H_ 6.76) to C-1′, C-2′ (δ_C_ 153.5), C-4′ positioned three O-substituents at C-1′, C-2′, and C-4′, HMBC correlations from H-5′ and the methoxy protons at 3.76 to C-7′ (δ_C_ 165.3) located a methyl ester at C-6′. The methoxy protons at 3.68 correlated to C-2′ (δ_C_ 153.5) in the HMBC spectrum, indicating it to be attached to C-2′.

A side-by-side comparison of the NMR data of **1** and **2** revealed that the two units were connected via the ether linkage C-2−O−C-1′, and the O-substituent at C-4′ was a hydroxy group. Thus, the structure of **1** was determined as shown in Figure 2.

**FIGURE 3:**
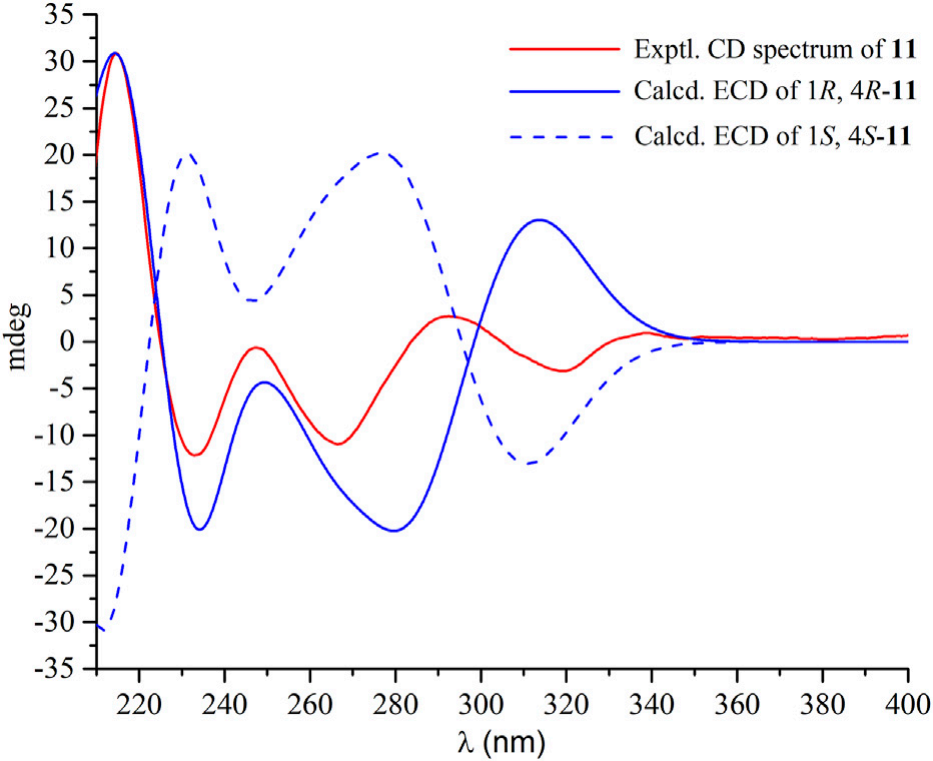
Experimental and calculated ECD spectrum of **11** in methanol.

Compound **11** had the molecular formula C_21_H_16_N_4_O_2_ as determined by the HRESIMS, indicating sixteen degrees of unsaturation. The ^1^H NMR spectrum (Supplementary Figure S26) displayed the resonances for two ABCD aromatic spin systems of two ortho-substituted benzene rings [δ_H_ 8.15 (dd, *J* = 8.0, 1.4 Hz), 7.81 (ddd, *J* = 8.0, 8.0, 1.6 Hz), 7.65 (*J* = 8.0 Hz), 7.54 (ddd, *J* = 8.0, 1.4 Hz), 7.40 (d, *J* = 8.0 Hz), 7.37 (d, *J* = 8.0 Hz), 7.11 (ddd, *J* = 8.0, 8.0, 1.2 Hz), 6.99 (dd, *J* = 8.0 Hz)]. A deshielded one-proton signal at 5.70 (t, *J* = 4.6, 2.8 Hz) was observed and was coupled to two isolated and geminally coupled doublets at δ_H_ 3.42 (1H, dd, *J* = 17.4, 2.8 Hz), 3.23 (1H, dd, *J* = 17.3, 4.6 Hz), indicating the presence of diastereotopic protons of a methylene unit next to a stereocenter and *α* to strong electronegative groups. A methyl singlet was found at δ_H_ 2.11 (3H s). The ^13^C NMR spectrum (Supplementary Figure S27) revealed the presence of 21 carbons, including two carbonyl carbons (δ_C_ 169.2, 159.4) and 12 aromatic carbons for two benzene rings. These structural features were very similar to those of *ent*-fumiquinazoline J (Zheng, et al., 2012), whose structure was elucidated by single-crystal X-ray diffraction using Mo Kα radiation. A comparison of their NMR data and specific rotation values confirmed the structure of **11** to be *ent*-fumiquinazoline J. A literature research revealed that the absolute configuration was resolved by comparing the optical rotation with that of a synthesized precursor of (−)-alantrypinone that had an identical gross structure (Hart and Magomedov, 1999). In the current study, the absolute configuration of **11** was confirmed to be 1*R* and 4*R* by comparisons of experimental electronic circular dichroism (ECD) with the calculated ECD spectra (Figure 3). The experimental and calculated ECD spectra exhibited similar curves, with positive Cotton effects at around 210 nm and negative Cotton effects at around 235 nm and 270 nm.

The other compounds were identified as monomethyl sulochrin (**3**) (Xu, et al., 2022), penipurdin A (**4**) (Xue, et al., 2015), questinol (**5**) (Said, et al., 2019), questin (**6**) (Said, Hou, Liu, Chao, Jiang, Zheng and Shao, 2019), emodin (**7**) (Meselhy, 2003), viridicatol (**8**) (Wei, et al., 2011), viridicatin (**9**) (Rudolf, et al., 2013), 3-*O*-methylviridicatin (**10**) (Guo, et al., 2023), aurantiomide C (**12**) (Xin, et al., 2007), 3′-(4-oxoquinazolin-3-yl)spiro [1H-indole-3,5′-oxolane]-2, 2′-dione (**13**) (Buttachon, et al., 2012), isochaetominine (**14**) (Huang, et al., 2016), asperfumigatin (**15**) (Xie, et al., 2015), pseurotin F2 (**16**) (Aoki, et al., 2004), 4(3H)-quinazolinone (**17**) (Wang, et al., 2011), 1-methyl-2(1H)-quinazolinone (**18**) (Liu, et al., 2022), didehydrobisdethiobis (methylthio)gliotoxin (**19**) (Zhang and An, 2019), *N*,*N*′-diphenylurea (**20**) (Zhang, et al., 2011), *p*-acetamidobenzoic acid (**21**) (Lewis, et al., 2003).

### 2.2 *Ent*-fumiquinazoline J (11) selectively inhibited cell proliferation in human hepatoma HepG2 cells

**FIGURE 4:**
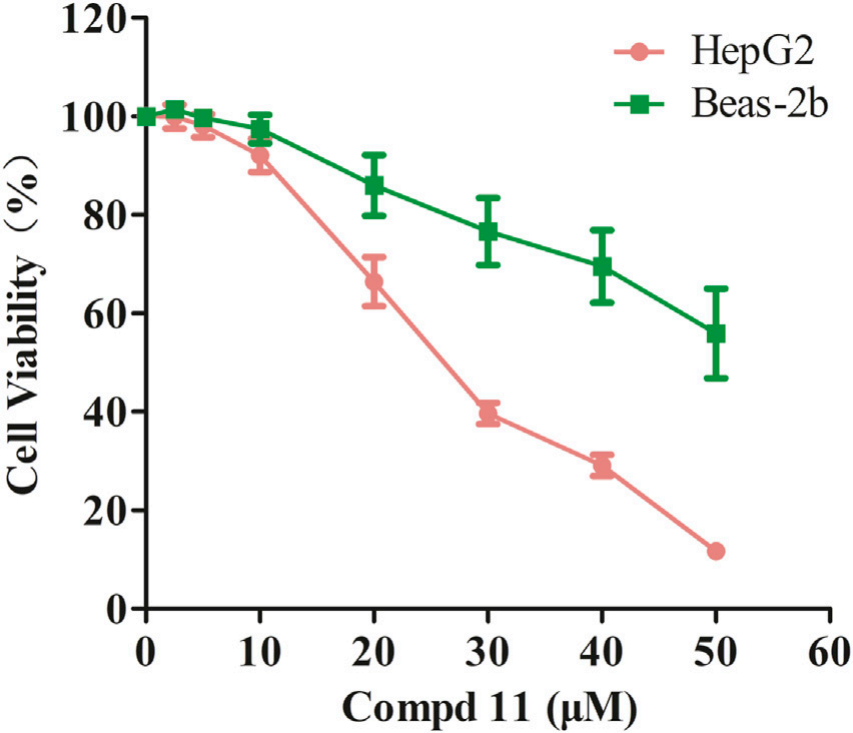
The inhibitory effect of compound **11** on cell proliferation in HepG2 and Beas-2b cells. HepG2 and Beas-2b cells were treated with different concentrations of compounds **11** for 48 h. Cell viability was measured by MTT assay. DMSO and Doxorubicin are used as negative control and positive control, respectively.

Initially, we evaluated the growth-inhibitory potencies of these compounds against a panel of cell lines at a concentration of 50 μM, including the human cancer cell lines HepG2, MDA-MB-231, SW620, and the normal human lung epithelial cell line Beas-2b. Only compounds **8** and **11** displayed inhibitory effects on cell proliferation with inhibition rates exceeding 50%, which were further evaluated to determine the IC_50_ values (Table 2). Compound **8** displayed moderate cytotoxicity against all tested cancer cell lines except HepG2, yet it also exhibited considerable toxicity towards normal cells, indicating a lack of selectivity. Additionally, the HepG2 cells were sensitive to compound **11**, with an IC_50_ value of 29.03 ± 0.4 μM, while it exhibited negligible cytotoxicity towards the normal human lung epithelial cells Beas-2b with IC_50_ values exceeding 50 μM (Table 2; Figure 4). These findings suggest that compound **11** exerts a preferential cytotoxic effect on human hepatoma HepG2 cells compared to normal cells.

**TABLE 2:** IC_50_ values (μM) of compounds **8** and **11** on cell proliferation.

No.	HepG2	MDA-MB-231	SW620	Beas-2b
**8**	>50	29.25 ± 0.8	24.53 ± 0.6	24.53 ± 0.6
**11**	29.03 ± 0.4	>50	>50	>50
Doxorubicin	6.3 ± 0.5	9.6 ± 0.6	2.2 ± 0.5	0.34 ± 0.3

### 2.3 Ent-fumiquinazoline J (11) inhibited migration and proliferation of HepG2 cells

**FIGURE 5:**
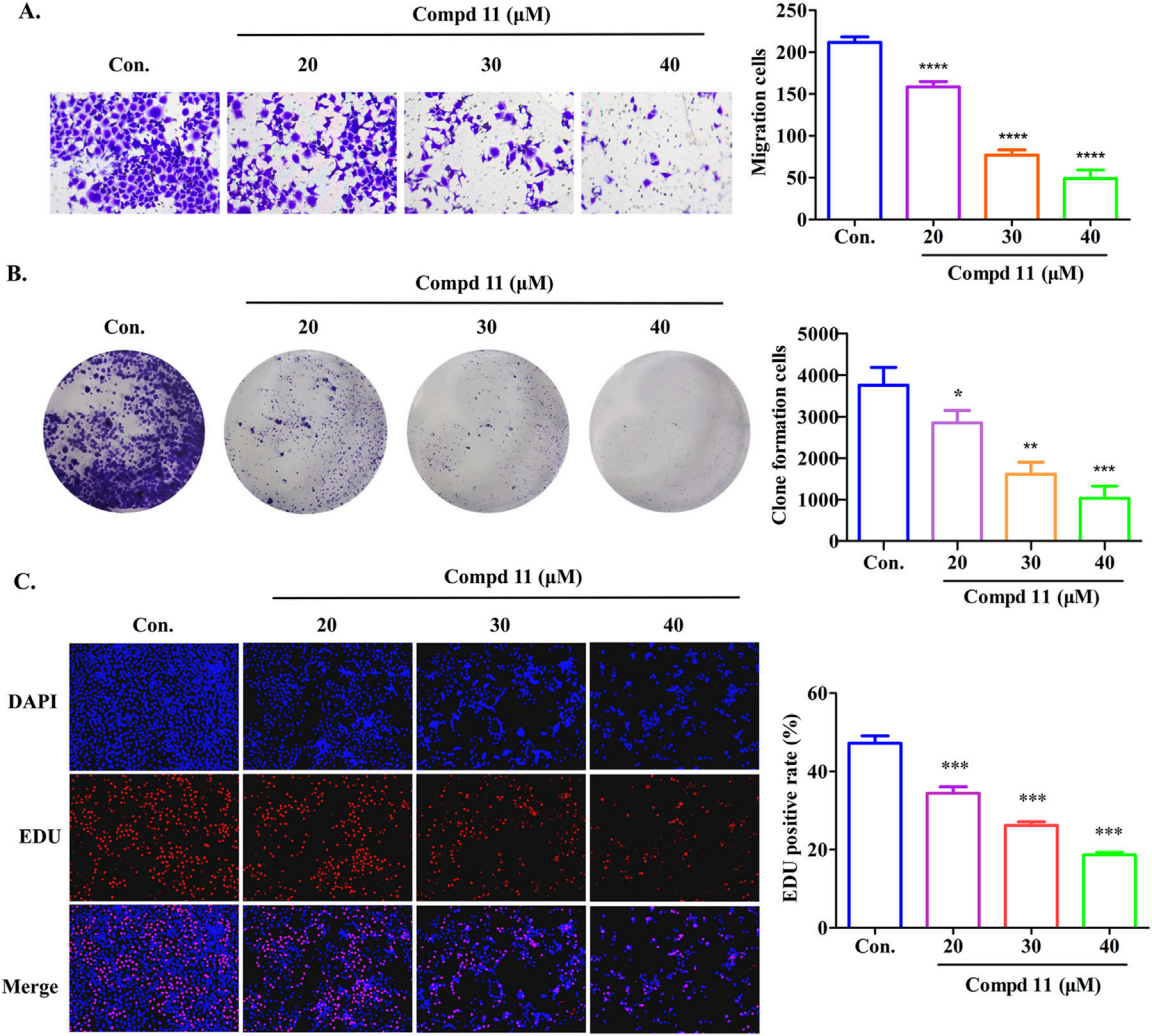
Compound **11** inhibits the migration and proliferation of HepG2 cells. HepG2 cells were treated with the indicated concentrations of compound **11** for 48 h, cell migration assay **(A)**, colony forming cell assay **(B)** and EDU staining **(C)** were performed. ****p < 0.0001, ***p < 0.001, **p < 0.01, and *p < 0.05 *versus* nontreated cells.

To assess the impact of compound **11** on the migratory capacity of HepG2 cells, transwell migration assays were conducted, accompanied by quantitative analysis. As depicted in Figure 5A, compound **11** potently inhibited the migratory potential of HepG2 cells in a dose-dependent fashion, as compared to the untreated controls. Moreover, the antiproliferative effects of compound **11** on HepG2 cells were substantiated by both colony formation and EDU incorporation assays. After a 48-h treatment with compound **11**, a significant and dose-dependent decrease in the number of colonies was observed. Notably, a marked reduction in colony-forming ability was evident at 20 μM, with a complete inhibition of colony formation observed at 30 μM and 40 μM (Figure 5B). Furthermore, the EDU incorporation assay was employed to evaluate the effects of compound **11** on DNA synthesis. Treatment with compound **11** resulted in a substantial decrease in EDU fluorescence intensity in HepG2 cells relative to untreated cells, indicating that compound **11** dose-dependently suppressed cellular proliferation (Figure 5C). In summary, treatment with compound **11** significantly inhibited cell migration, colony formation, and DNA synthesis in HepG2 cells.

### 2.4 Ent-fumiquinazoline J (11) induced cell apoptosis and cycle arrest of HepG2 cells

**FIGURE 6:**
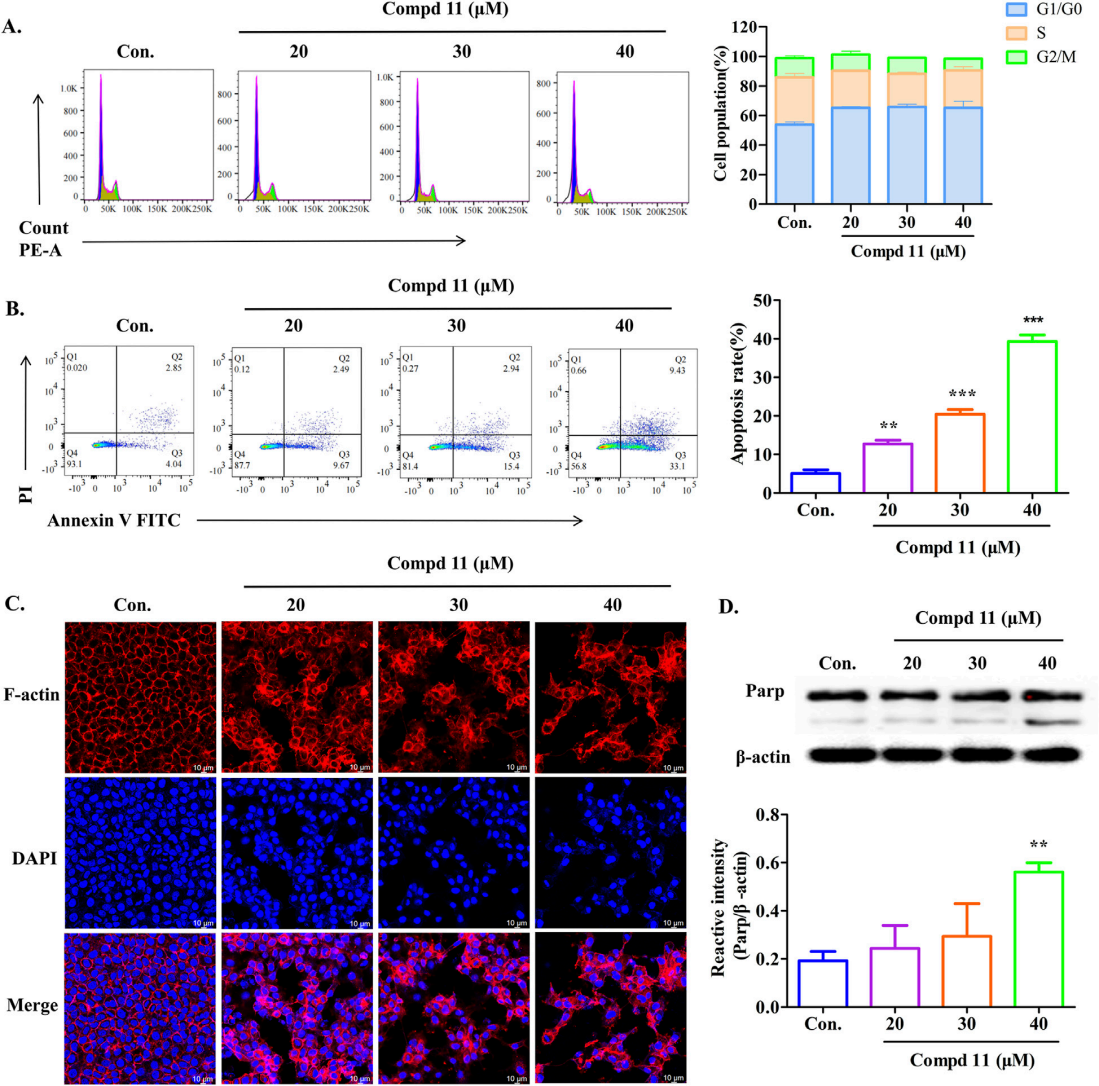
Apoptosis and cell cycle arrest of HepG2 cells induced by compound **11**. HepG2 cells were incubated with compound **11** (20, 30, 40 μM) for 48 h. **(A)** Cells were stained with propidium iodide (PI), and cell cycle distribution was assessed by flow cytometry. **(B)** Flow cytometric analysis of apoptosis and quantitative analysis of the ratio of early apoptotic cells were measured. Fluorescence quantitative analysis. **(C)** Immunofluorescence studies on HepG2 cells’ structure (63X) after treatment with compound **11** for 48 h. DAPI for the detection of the nuclei staining (blue) and actin filaments were labeled with phalloidin (red, the scale bar expresses 100 μm. **(D)** Western blotting analysis was conducted to determine the expression of Parp protein levels. β-actin was used as a loading control. ***p < 0.001 and **p < 0.01 *versus* nontreated cells.

To investigate whether the growth-inhibitory effects of compound **11** were associated with cell cycle arrest, we analyzed the cell cycle distribution of HepG2 cells using flow cytometry. As illustrated in Figure 6A, treatment with different concentrations of compound **11** for 48 h led to a significant increase in the proportion of HepG2 cells in the G0/G1 phase, coupled with a decrease in the G2/M phase population. Specifically, the percentage of cells in the G0/G1 phase increased to 65.24% at a concentration of 20 μM, compared to 53.835% in the control group, which may be indicative of an early apoptotic response. Additionally, we conducted a two-color flow cytometry analysis using Annexin V FITC/PI double-staining and observed a slight increase in the apoptotic cell population upon treatment with compound **11**. After 48 h of treatment with 20, 30, and 40 µM compound **11**, the percentages of early apoptotic cells increased dose-dependently to 12.7%, 20.4%, and 39.3%, respectively, as compared to 5.1% in the control group (Figure 6B). These findings suggest that compound **11** elicits cytotoxicity partially through the induction of apoptosis.

Furthermore, the organization of the F-actin cytoskeleton in HepG2 cells was visualized in Figure 6C. Untreated HepG2 cells displayed a typical F-actin cytoskeletal structure. However, after 48 h of exposure to compound **11**, significant alterations in cytoskeletal organization were observed, resulting in aberrant cell morphology and a decrease in F-actin density. Additionally, we assessed the levels of poly (ADP-ribose) polymerase (PARP) cleavage. Notably, compound **11** induced PARP cleavage in HepG2 cells at a concentration of 40 µM (Figure 6D). Collectively, these results indicate that compound **11** can induce cell cycle arrest at the G0/G1 phase and may eventually lead to apoptosis, potentially mediated by PARP cleavage, at concentrations exceeding the IC_50_ value for HepG2 cells.

### 2.5 Ent-fumiquinazoline J (11) triggers paraptosis in HepG2 cells

**FIGURE 7:**
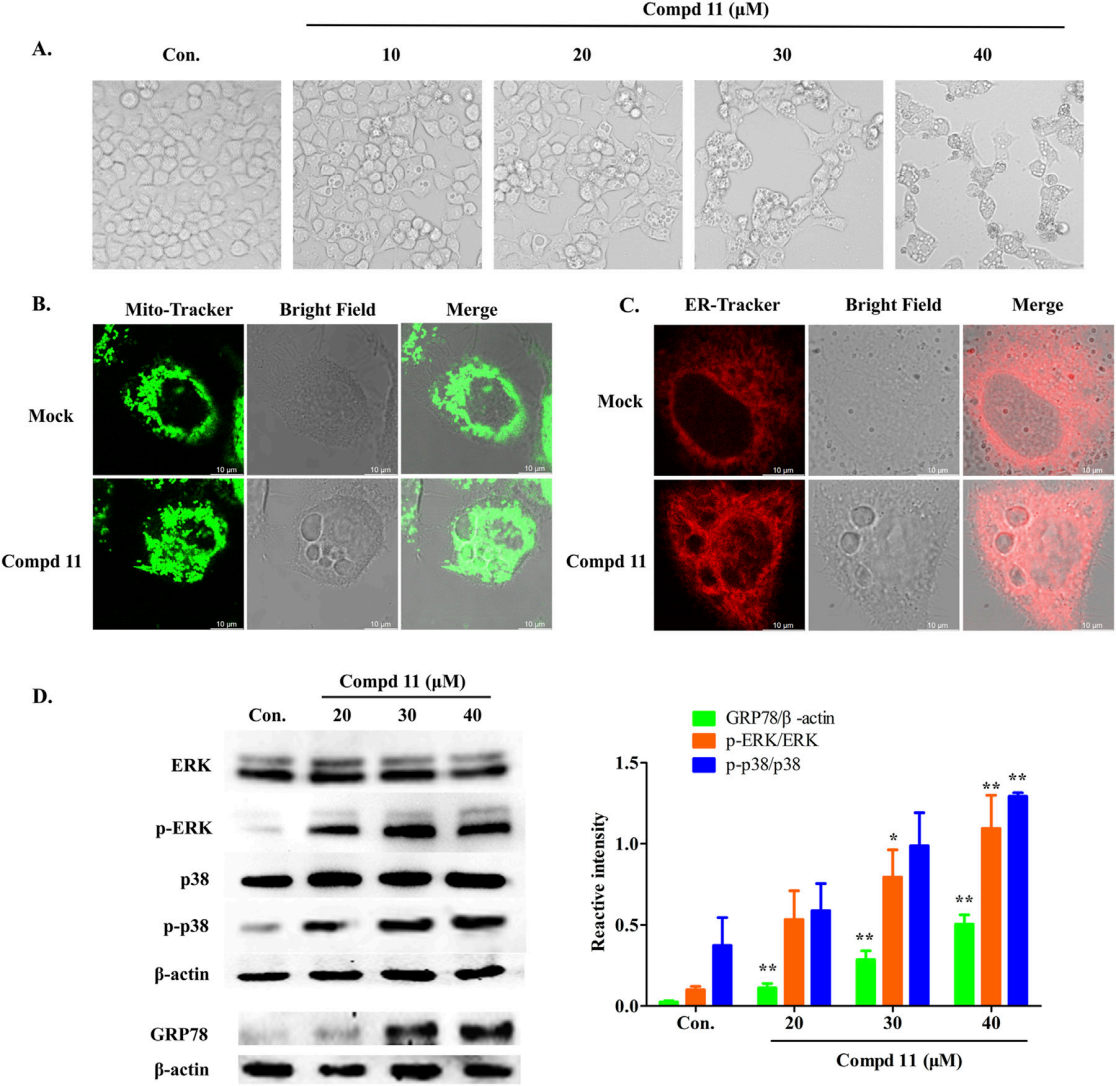
Compound **11** induced paraptosis, mitochondria and endoplasmic reticulum (ER) stress. **(A)** HepG2 cells were treated with compound **11** (10, 20, 30, 40 μM) for 48 h and cellular morphological were observed by phase-contrast microscopy. **(B)** The cells were treated with 30 μM of compound **11** for 48 h and stained with Mito tracker buffer by laser scanning confocal microscopy. The scale bar expresses 10 μm. **(C)** The cells were incubated with compound **11** (30 μM) for 48 h and stained with ER tracker buffer. The scale bar expresses 10 μm. **(D)** Western blotting analysis of the expression of ERK, p-ERK, p38, p-p38 and GRP78 after compound **11** treated with the indicated concentrations of CBD for 24 h β-actin is shown as a loading control, ***p < 0.001, **p < 0.01, and *p < 0.05 *versus* nontreated cells.

In the course of this study, we noted that after 48 h of exposure to compound **11**, a significant number of vacuoles emerged within the HepG2 cells (Figure 7A). With increasing concentrations of compound **11**, the quantity of vacuoles within the cells progressively augmented, albeit with a concomitant reduction in the size of the vacuoles. Concurrently, cellular morphological alterations such as cell shrinkage, cytoplasmic retraction, and nuclear chromatin condensation were apparent (Figure 7A). The occurrence of vacuolation-associated cell death led us to investigate the potential involvement of paraptosis, a form of cell death characterized by the dilation of the endoplasmic reticulum (ER) and mitochondria (Liu, et al., 2021; Xue, et al., 2018). Accordingly, ER and mitochondrial staining assays were performed. Laser scanning confocal microscopy revealed that the vacuoles were distinctly positive for ER-Tracker Red and Mito-Tracker Red, indicating that treatment with compound **11** induced vacuolation originating from both the ER and mitochondria (Figures 7B, C). These findings collectively suggest that compound **11** induced ER vacuolation with characteristics typical of paraptosis. The formation of ER vacuoles is often linked to sustained ER stress. Hence, we hypothesized that the vacuolation induced by compound **11** might lead to the onset of persistent ER stress. Indeed, the expression of GRP78, a pivotal indicator of the ER stress response, was markedly upregulated following treatment with compound **11** (Figure 7D).

Activation of the mitogen-activated protein kinase (MAPK) pathway is frequently observed in cells undergoing apoptosis and paraptosis (Li, et al., 2022; Wang, et al., 2021; Yue and López, 2020). To explore the impact of compound **11** on various MAPK pathways in HepG2 cells, we conducted a series of assays. As depicted in Figure 7D, the phosphorylation of p38 and ERK was elevated in response to treatment with compound **11**, without alterations in the total protein levels of p38 and ERK. This indicates that compound **11** activated the p38 and ERK pathways, potentially contributing to the induction of apoptosis and paraptosis.

## 3 Materials and methods

### 3.1 General experimental procedure

Specific rotations were measured using an SGW^®^-1 automatic polarimeter (Shanghai Jing Ke Industrial Co., Ltd.). Ultraviolet (UV) spectra were recorded on a UV-2600 spectrometer (Shimadzu). ECD spectra were measured on an Applied Photophysics Chirascan spectrometer. The NMR spectra were acquired on a Bruker Avance III HD-400 NMR spectrometer using TMS as a reference. HRESIMS spectra were obtained on an agilent 6546 LC/Q-TOF spectrometer fitted with an ESI source. Semi-preparative high-performance liquid chromatography (HPLC) was undertaken on a waters 2,535 pump equipped with waters 2998 PDA detector. The separation was carried out on a YMC-Pack ODS-A HPLC column (semipreparative, 250 × 10 mm, S-5 μm, 12 nm). Silica gel (300–400 mesh, Qingdao Haiyang Chemical Co., Ltd.), C18 reversed-phase silica gel (12 nm, S-50 μm, YMC Co., Ltd.), and MCI gel (CHP20P, 75–150 μm, Mitsubishi Chemical Industries Ltd.) were used for column chromatography (CC). All solvents were of analytical grade (Guangzhou Chemical Reagents Company, Ltd.). DMEM, L15 medium, and fetal bovine serum (FBS) were purchased from Thermo Scientific HyClone. The anti-PARP, anti-ERK, anti-p-ERK, and anti-GPR78 antibodies were sourced from Cell Signaling Technology. The anti-p-p38 and anti-p38 antibodies were obtained from Affinity Biosciences LTD. Anti-β-actin and secondary antibodies were purchased from Proteintech.

### 3.2 Fungal strain and identification

The fungal strain was isolated from wild marine oysters and identified as *A. fumigatus* by morphological analysis of the fungal mycelium and a BLAST comparison of its rDNA internal transcribed spacer (ITS) sequence in GenBank (http://www.ncbi.nlm.nih.gov). The I'T'S sequence was deposited in GenBank (accession number: PQ203384).

### 3.3 Fermentation, extraction, and isolation

The fermentation was performed in 100 Erlenmeyer flasks (500 mL), each containing 80 g of rice and 100 mL of filtered seawater. The contents were soaked for 1 h before autoclaving for 20 min at 15 psi. Each flask was inoculated with 1.0 mL of the spore inoculum and incubated at room temperature (r. t.) for 30 days. The fermented materials were extracted with EtOAc (3 × 10,000 mL) at 25°C for 30 min. After vacuum evaporation, the EtOAc extract (21.0 g) was fractionated on a silica gel eluting with CH_2_Cl_2_/MeOH (100: 1, 50: 1, 25: 1, 12.5: 1, 5: 1, 1: 1 and MeOH) to afford 6 fractions (Fr.A–Fr.G). Fraction Fr. A was separated into five fractions (A-1−A-5) by ODS column chromatography (CC) with a gradient from 25:75 to 100:0 (MeOH/H_2_O). A-2 was subjected to Sephadex LH-20 with CH_2_Cl_2_/MeOH (1:1) as solvent to obtain three fractions (A-2-1−A-2-3). A-2-2 was further separated and purified by C18 HPLC (MeOH/H_2_O = 1:1, 2 mL/min) to give compound **6** (5.7 mg). A-5 was separated by C18 HPLC (MeOH/H_2_O = 50:50, 2 mL/min) to obtain four subfractions (A-5-1, A-5-2, A-5-3 and A-5-4). A-5-3 was subjected to Sephadex LH-20 (CH_2_Cl_2_/MeOH = 1:1) to obtain A-5-3-1 and A-5-3-2. A-5-3-1 was separated and purified by Semi-HPLC (AcCN/H_2_O = 37:63, 2 mL/min) to obtain compound **12** (3.2 mg). Fr. D was purified by Sephadex LH-20 (CH_2_Cl_2_/MeOH = 1:1) to give compound **18** (12 mg). Fr. E was recrystallized from CH_2_Cl_2_/MeOH (1:1) to obtain compound **3** (2.23 g). Fr.B was separated by ODS CC with a gradient of MeOH/H_2_O (25%→100%) to obtain ten subfractions (B-1−B-10). B-2 was further separated by C18 HPLC (MeOH/H_2_O = 45:55%, 2 mL/min) to obtain B-2-1, B-2-2, and compound **21** (9 mg). Compounds **16** (7 mg) and **17** (6 mg) were obtained by purification of B-2-2 on C18 HPLC (AcCN/H_2_O = 37:63%, 2 mL/min). Fr.B-4 was separated by C18 HPLC (MeOH/H_2_O = 55:45%, 2 mL/min) to obtain B-4-1, compound **2** (10 mg), and compound **19** (5 mg). Fr.B-4-1 was further chromatographed on a Sephadex LH-20 (CH_2_Cl_2_/MeOH = 1:1) to obtain compound **14** (15 mg). Fr.B-5 was subjected to Sephadex LH-20 (CH_2_Cl_2_/MeOH = 1:1) to obtain B-5-1, B-5-2, B-5-3 and compound **8** (8 mg). Fr.B-5-1 was separated by C18 HPLC (MeOH/H_2_O = 55:45%, 2 mL/min) to afford compounds **13** (5 mg) and **5** (2 mg). Fr.B-5-3 was separated by C18 HPLC (MeOH/H_2_O = 53:47%, 2 mL/min) to obtain compound **1** (12 mg). Fr.B-6 was purified by Sephadex LH-20 (CH_2_Cl_2_/MeOH = 1:1) to obtain B-6-1, B-6-2, and compound **20** (3 mg). Fr.B-6-1 was separated by C18 HPLC (MeOH/H_2_O = 45:55%, 2 mL/min) to obtain compound **4** (4 mg). Fr.B-7 was subjected to Sephadex LH-20 (CH_2_Cl_2_/MeOH = 1:1) to obtain four subfractions (B-7-1, B-7-2, B-7-3 and B-7-4). B-7-1 was separated by C18 HPLC (MeOH/H_2_O = 69:31%, 2 mL/min) to obtain compounds **11** (6 mg) and **10** (3 mg). Fr.B-7-2 was separated by C18 HPLC (MeOH/H_2_O = 69:31%, 2 mL/min) to obtain compound **15** (4 mg). B-7-3 was isolated by C18 HPLC (MeOH/H_2_O = 70:30%, 2 mL/min) to obtain compound **9** (3 mg). Fr.B-9 was isolated by C18 HPLC (MeOH/H_2_O = 73:27%, 2 mL/min) to obtain compound **7** (16 mg).

7-Ethyl circinophoric acid (**1**): Yellow powder; UV (MeOH) λ_max_ (log ε) 205 (4.53), 281 (3.68) nm; ^1^H and ^13^C NMR data, see Table 1; HRESIMS *m*/*z* 413.1235 [M + Na]^+^ (calcd for C_20_H_22_O_8_Na^+^, 413.1212).

*Ent*-fumiquinazoline J (**11**): Yellow powder; [*α*]^20^_D_ −107 (*c* 0.1, MeOH); ECD (*c* 0.10 mM, MeOH) *λ*_max_ (mdeg) 267 (−10.9), 233 (−12.1), 214 (+30.9) nm; HRESIMS *m*/*z* 357.1377 [M + Na]^+^ (calcd for C_21_H_17_N_4_O_2_^+^, 357.1346). ^1^H NMR (400 MHz, DMSO-*d*_6_): δ_H_ 11.24 (1H, s, H-19), 9.60 (1H, s, H-2), 8.15 (1H, dd, *J* = 8.0, 1.4 Hz, H-8), 7.81 (1H, ddd, *J* = 8.0, 8.0, 1.4 Hz, H-10), 7.65 (1H, d, *J* = 8.0 Hz, H-), 7.54 (1H, ddd, *J* = 8.0, 1.4 Hz, H-24), 7.40 (1H, d, *J* = 8.0 Hz, H-21), 7.37 (1H, d, *J* = 8.0 Hz, H-24), 7.11 (1H, ddd, *J* = 8.0, 8.0, 1.4 Hz, H-22), 6.99 (1H, dd, *J* = 8.0 Hz, H-23), 5.70 (1H, dd, *J* = 4.6, 2.8 Hz, H-4), 3.42 (1H, dd, *J* = 17.4, 2.8 Hz, H-15a), 3.23 (1H, dd, *J* = 17.3, 4.6 Hz, H-15b), 2.11 (3H, s, H-16). ^13^C NMR (100 MHz, DMSO-*d*_6_): 18.3 (C-16), 25.6 (C-15), 54.2 (C-4), 54.6 (C-1), 105.6 (C-17), 111.7 (C-24), 118.2 (C-21), 119.4 (C-24), 120.2 (C-7), 122.3 (C-22), 126.4 (C-8), 127.3 (C-11), 127.4 (C-9), 127.4 (C-11), 134.0 (C-18), 134.8 (C-10), 134.9 (C-20), 146.7 (C-12), 154.4 (C-14), 159.4 (C-6), 169.2 (C-3).

### 3.4 ECD calculation

Conformational analyses of 1*R*, 4*R*-**11** were carried out via random searching in the Sybyl-X 2.0 using the MMFF94S force field with an energy cutoff of 3.0 kcal/mol. Due to the rigid structure. The result showed only one conformer for 1*R*, 4*R*-**11**. The conformer was re-optimized using density functional theory (DFT) at the B3LYP/6-31+G (d,p) level in MeOH using the solvation model density (SMD) by the GAUSSIAN 09 program. The energies, oscillator strengths, and rotational strengths (velocity) of the first 30 electronic excitations were calculated using the TDDFT methodology at the B3LYP/6-31+G (d,p) level in MeOH. The ECD spectrum was simulated by the overlapping Gaussian function (half the bandwidth at 1/e peak height, σ = 0.25 eV, UV correction = 7 nm). Theoretical ECD spectrum of the corresponding enantiomer 1*S*, 4*S*-11 were obtained by directly inverting the ECD spectrum of 1*R*, 4*R*-**11**.

### 3.5 Cell culture

Human cancer HepG2, MDA-MB-231, SW620, and Beas-2b cells were obtained from the Shanghai Cell Bank, Chinese Academy of Sciences. HepG2, MDA-MB-231, and Beas-2b cells were maintained in DMEM medium, whereas SW620 cells were cultured in Leibovitz’s L15 medium supplemented with 10% inactivated FBS and 1% penicillin/streptomycin at 37°C in a humidified atmosphere with 5% CO_2_.

### 3.6 Cell viability assay

The cytotoxic effect of all compounds on human cancer cell lines was evaluated using the 3-(4,5-dimethylthiazol-2-yl)-2,5-diphenyltetrazolium bromide (MTT) assay. HepG2, MDA-MB-231, SW620, and Beas-2b cells were seeded into a 96-well plate at a density of 4 × 10^4^ cells per well. Cells were treated with varying concentrations of compounds (0, 2.5, 5, 10, 20, 30, 40, and 50 μM) for 48 h. Following the treatment, MTT solution (1 mg/mL) was added to each well. After a 2-h incubation, the MTT solution was removed, and the cells were solubilized with DMSO. Absorbance was measured at 490 nm using a Multifunction microplate reader (Bio Tek).

### 3.7 Colony formation assay

HepG2 cells in the logarithmic growth phase were seeded into 6-well culture plates at a density of 2000 cells per well for each experimental group and treated with various doses of compound **11** (0, 20, 30, and 40 μM) for 7 days. After the colonies had formed, the cells were imaged under a microscope. The plates were then washed with ddH_2_O, fixed with 4% paraformaldehyde, and stained with crystal violet solution. The colony formation efficiency was subsequently calculated.

### 3.8 Cell migration assay

The cells were cultured to the logarithmic growth phase and a cell suspension containing 2×10^4^ cells was added to the upper chamber of a Transwell insert. The lower chamber was filled with medium containing 20% FBS. The cells were treated with the indicated concentrations of compound **11** (0, 20, 30, and 40 μM) for 48 h. After incubation, the Transwell insert was carefully removed, and the non-migrated cells in the upper chamber were gently swabbed away using a cotton swab. The cells that had migrated to the lower chamber were fixed with 4% paraformaldehyde and stained with crystal violet. Excess dye was removed by washing with ddH_2_O, and the cells were allowed to dry before being observed and photographed under a microscope. Statistical analysis was performed on the collected data.

### 3.9 EDU staining assay

Cells in the logarithmic growth phase were seeded into a 12-well culture plate at a density of 5×10^5^ cells per well. Cells were then treated with various concentrations of compound **11** (0, 20, 30, and 40 μM) for 48 h. EDU was diluted to a concentration of 20 μM in complete medium and added to the cell culture plate, followed by incubation at 37°C for 2 h. The medium was then removed, and the cells were fixed with 4% paraformaldehyde at room temperature for 30 min. After fixation, the cells were washed and incubated with a permeabilization agent. The staining reaction was initiated by adding the reaction mixture, and the cells were incubated in the dark. Nuclei were stained using Hoechst 33,342. EDU-labeled cells were observed under a fluorescence microscope, photographed, and counted.

### 3.10 Annexin-V/PI staining assay

Cell apoptosis was detected using the Annexin-V FITC/PI Apoptosis Detection Kit (Beyotime) according to the manufacturer’s instructions. Cells were treated with the indicated concentrations of compound **11** (0, 20, 30, and 40 μM) for 48 h, and then stained with fluorescein isothiocyanate-tagged Annexin-V antibodies and propidium iodide (PI) for 15 min. The rate of apoptotic cells was detected by the BD FACS Canto II Flow Cytometer (BD Biosciences).

### 3.11 Cell cycle analysis

Cells were seeded on a six-well plate and treated with different concentrations of compound **11** for 48 h. Post-treatment, cells were harvested, fixed, and stored in ethanol at 4°C overnight. Cell cycle was analyzed according to the manufacturer’s instructions. The stained cells were analyzed by the BD FACS Canto II Flow Cytometer (BD Biosciences).

### 3.12 Immunofluorescence assay

HepG2 cells in the logarithmic growth phase were seeded into 12-well plates and treated with 20, 30, and 40 μM of compound **11** for 48 h. Cells were then fixed with 4% paraformaldehyde for 20 min. After 0.1% Triton X-100 treatment, cells were blocked with 5% bovine serum albumin (BSA) and incubated with 200 μL of rhodamine-labeled phalloidin (50 μg/mL) at room temperature in the dark for 30 min. DAPI was used to stain the nuclei, and the cover glass was mounted with fluorescent mounting medium. The stained cells were detected and analyzed for F-actin using a laser confocal microscope (Leica).

### 3.13 Western blotting analysis

Cells were seeded in 60 mm cell culture dishes and treated with 20, 30, and 40 μM of compound **11** for 48 h. Post-treatment, cells were harvested and lysed under cold conditions, followed by estimation of protein concentration using the Bradford assay. Proteins were separated by SDS-PAGE and then transferred onto PVDF membranes. The membranes were blocked with 5% BSA solution for 1 h and incubated overnight at 4°C with primary antibodies against Parp, ERK, p-ERK, p38, p-p38, GPR78, and β-actin, respectively. After washing, the membranes were incubated with secondary antibodies at room temperature for 1 h. Chemiluminescence signals were detected using a multifunctional imaging system (Thermo). Gray scale analysis of protein bands was performed using ImageJ software (NIH).

### 3.14 Mitochondria staining assay

HepG2 cells were incubated with 30 μM of compound **11** for 48 h. Mitochondria staining was performed according to the manufacturer’s procedure. Stained cells were detected using a laser confocal microscope (Leica).

### 3.15 ER staining assay

HepG2 cells were incubated with 30 μM of compound **11** for 48 h and then treated with ER Tracker Red (1 μM) for 25 min. ER staining was performed according to the manufacturer’s procedure. ER-stained cells were detected using a laser confocal microscope (Leica).

### 3.16 Statistical analysis

All values are presented as the mean ± standard deviation. Statistical analysis was conducted using GraphPad Prism software (GraphPad Software) and a student unpaired t-test was performed. Significance was indicated as follows: ****p < 0.0001, ***p < 0.001, **p < 0.01, and *p < 0.05 *versus* nontreated cells. All experiments were replicated at least three times.

## 4 Discussion and conclusion

**FIGURE 8:**
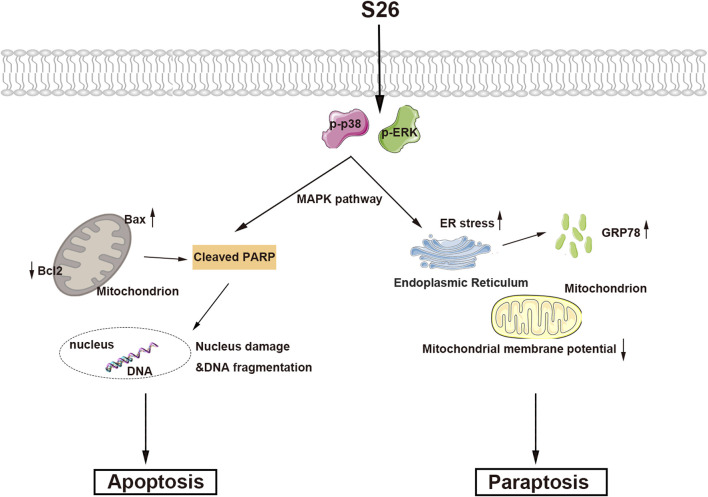
Mechanistic overview of *ent*-fumiquinazoline J’s dual-induced cell death mechanism.

Cancer remains a leading cause of mortality worldwide, and the induction of tumor cell death has long been a central strategy in cancer therapy. Traditional approaches have primarily focused on apoptosis, a form of programmed cell death (PCD). However, the discovery of various forms of PCD, including necroptosis, autophagy, ferroptosis, copper death, pyroptosis, and paraptosis, has expanded our understanding of cell death mechanisms and their potential as therapeutic targets. Among these, paraptosis stands out as a distinct form of PCD characterized by mitochondrial or endoplasmic reticulum swelling and cytoplasmic vacuolization in the absence of classical apoptotic markers. The discovery of paraptosis by Sperandio et al., in 2000 (Sperandio, et al., 2000) opened new avenues for cancer therapy, particularly for tumors that have developed resistance to conventional apoptosis-inducing agents. Recently, an increasing body of research indicates that numerous natural compounds induce tumor cell death through the pathway of paraptosis (Li, Zhao, Wang, Luo, Shi, Yan, Li, Liu, Yang, Tan, Zhang, Chen, Lai, Huang, Zhou, Ma, Fang and Gao, 2022; Su, et al., 2022). Our study presents compound **11**, a rare alkaloid isolated from marine fungi, which selectively inhibits the proliferation of HepG2 cells by inducing both apoptosis and paraptosis (Figure 8). This dual action is significant as it circumvents the resistance mechanisms often seen in cancer cells that are targeted by single-modality therapies. The activation of apoptotic-related protein PARP and the increase in the endoplasmic reticulum stress protein GPR78, along with cytoplasmic vacuolization, suggest that compound **11** induces paraptosis through the disruption of endoplasmic reticulum homeostasis. Furthermore, the concentration-dependent increase in p-p38 and p-ERK1/2 protein levels indicates that compound **11** may mediate its effects through the MAPK signaling pathway, a known regulator of cell death and survival. The ability of compound **11** to induce paraptosis in HepG2 cells highlights its potential as a novel therapeutic agent for liver cancer. The MAPK pathway, a critical cellular signaling mechanism, has been implicated in various cellular processes, including cell growth, differentiation, and death. Targeting this pathway with compound **11** could provide a new strategy for treating liver cancer, especially for patients with drug-resistant tumors. The discovery and characterization of compound **11** exemplify the untapped potential of marine natural products in cancer therapy.

In summary, our investigation of the marine-derived fungus *A. fumigatus* YB4-17, sourced from marine oysters, resulted in the isolation of seven polyketides (**1**-**7**) and fourteen alkaloids (**8**-**21**), including a novel diphenylether derivative (**1**). The structures were elucidated using comprehensive spectroscopic analysis, including 1D and 2D NMR spectroscopy and HRESIMS data. The in-depth biological evaluation of the isolated compounds revealed that compound **11** possesses significant cytotoxic selectivity, preferentially inhibiting the proliferation of HCC HepG2 cells while exhibiting negligible toxicity to normal cells. Furthermore, our findings underscore the therapeutic potential of compound **11** as a dual inducer of apoptosis and paraptosis in HepG2 cells. It should be highlighted that our predictive targeting for compound 11 is still in its infancy, yielding results that, though not definitive, establish a foundation for forthcoming research endeavors. Future studies are warranted to fully delineate the molecular underpinnings of compound **11**s action and to explore its synergistic potential when integrated with current cancer therapeutic regimens. Our study opens new horizons for marine natural products in the development of novel and effective cancer therapeutics.

## Data Availability

The datasets presented in this study can be found in online repositories. The names of the repository/repositories and accession number(s) can be found in the article/Supplementary Material.
