# Supplementary material for: Polyketides and alkaloids from the fungus Aspergillus Fumigatus YB4-17 and ent-Fumiquinazoline J induce apoptosis, paraptosis in human hepatoma HepG2 cells
Source: Front Pharmacol. 2024 Dec 4;15:1487977. doi: 10.3389/fphar.2024.1487977 (PMC11667090; doi:10.3389/fphar.2024.1487977)
Supplement: Supplementary file 1 [file DataSheet1.DOCX]

**Supporting Information**

Polyketides and Alkaloids from the Fungus *Aspergillus Fumigatus* YB4-17 and Ent-Fumiquinazoline J Induce Apoptosis, Paraptosis in Human Hepatoma HepG2 Cells

Huannan Wang ^1,†^, Lixiang Sun ^1,3,†^, Xueyang Ma ^1,3^, Shihao Jin ^1^, Chunmei Sai ^1^, Maocai Yan ^1^, Zhongbin Cheng ^2,*^, and Zhen Zhang ^1,*^

^1^ *School of Pharmacy, Jining Medical University, 669 Xueyuan Road, Rizhao 276800, Shandong, China;*

^2^ *Key Laboratory of Tropical Biological Resources of Ministry of Education, School of Pharmaceutical Sciences, Hainan University, Haikou 570228, People's Republic of China;*

^3^ *School of Pharmacy, Binzhou Medical University, 346 Guanhai Road, Yantai 264003, Shandong, China;*

| **Table of Contents** | **Page** |
| --- | --- |
| **Figure S1:**^1^H NMR Spectrum of **1** in DMSO-*d*_6_ (400 MHz) | 1 |
| **Figure S2:** ^13^C NMR Spectrum of **1** in DMSO-*d*_6_ (100 MHz) | 1 |
| **Figure S3**: HSQC Spectrum of **1** in DMSO-*d*_6_ | 2 |
| **Figure S4:** COSY Spectrum of **1** in DMSO-*d*_6_ | 2 |
| **Figure S5**: HMBC Spectrum of **1** in DMSO-*d*_6_ | 3 |
| **Figure S6**: NOESY Spectrum of **1** in DMSO-*d*_6_ | 3 |
| **Figure S7**: HRESIMS Spectrum of **1** | 4 |
| **Figure S8:**^1^H NMR Spectrum of **2** in DMSO-*d*_6_ (400 MHz) | 4 |
| **Figure S9:**^13^C NMR Spectrum of **2** in DMSO-*d*_6_ (100 MHz) | 5 |
| **Figure S10:**^1^H NMR Spectrum of **3** in DMSO-*d*_6_ (400 MHz) | 5 |
| **Figure S11:** ^13^C NMR Spectrum of **3** in DMSO-*d*_6_ (100 MHz) | 6 |
| **Figure S12:**^1^H NMR Spectrum of **4** in DMSO-*d*_6_ (400 MHz) | 6 |
| **Figure S13:** ^13^C NMR Spectrum of **4** in DMSO-*d*_6_ (100 MHz) | 7 |
| **Figure S14:**^1^H NMR Spectrum of **5** in DMSO-*d*_6_ (400 MHz) | 7 |
| **Figure S15:** ^13^C NMR Spectrum of **5** in DMSO-*d*_6_ (100 MHz) | 8 |
| **Figure S16:**^1^H NMR Spectrum of **6** in DMSO-*d*_6_ (400 MHz) | 8 |
| **Figure S17:** ^13^C NMR Spectrum of **6** in DMSO-*d*_6_ (100 MHz) | 9 |
| **Figure S18:**^1^H NMR Spectrum of **7** in DMSO-*d*_6_ (400 MHz) | 9 |
| **Figure S19:** ^13^C NMR Spectrum of **7** in DMSO-*d*_6_ (100 MHz) | 10 |
| **Figure S20:**^1^H NMR Spectrum of **8** in DMSO-*d*_6_ (400 MHz) | 10 |
| **Figure S21:** ^13^C NMR Spectrum of **8** in DMSO-*d*_6_ (100 MHz) | 11 |
| **Figure S22:**^1^H NMR Spectrum of **9** in DMSO-*d*_6_ (400 MHz) | 11 |
| **Figure S23:** ^13^C NMR Spectrum of **9** in DMSO-*d*_6_ (100 MHz) | 12 |
| **Figure S24:**^1^H NMR Spectrum of **10** in DMSO-*d*_6_ (400 MHz) | 12 |
| **Figure S25:** ^13^C NMR Spectrum of **10** in DMSO-*d*_6_ (100 MHz) | 13 |
| **Figure S26:**^1^H NMR Spectrum of **11** in DMSO-*d*_6_ (400 MHz) | 13 |
| **Figure S27:** ^13^C NMR Spectrum of **11** in DMSO-*d*_6_ (100 MHz) | 14 |
| **Figure S28:**^1^H NMR Spectrum of **12** in DMSO-*d*_6_ (400 MHz) | 14 |
| **Figure S29:** ^13^C NMR Spectrum of **12** in DMSO-*d*_6_ (100 MHz) | 15 |
| **Figure S30:**^1^H NMR Spectrum of **13** in DMSO-*d*_6_ (400 MHz) | 15 |
| **Figure S31:** ^13^C NMR Spectrum of **13** in DMSO-*d*_6_ (100 MHz) | 16 |
| **Figure S32:**^1^H NMR Spectrum of **14** in DMSO-*d*_6_ (400 MHz) | 16 |
| **Figure S33:** ^13^C NMR Spectrum of **14** in DMSO-*d*_6_ (100 MHz) | 17 |
| **Figure S34:**^1^H NMR Spectrum of **15** in DMSO-*d*_6_ (400 MHz) | 17 |
| **Figure S35:** ^13^C NMR Spectrum of **15** in DMSO-*d*_6_ (100 MHz) | 18 |
| **Figure S36:**^1^H NMR Spectrum of **16** in DMSO-*d*_6_ (400 MHz) | 18 |
| **Figure S37:** ^13^C NMR Spectrum of **16** in DMSO-*d*_6_ (100 MHz) | 19 |
| **Figure S38:**^1^H NMR Spectrum of **17** in DMSO-*d*_6_ (400 MHz) | 19 |
| **Figure S39:** ^13^C NMR Spectrum of **17** in DMSO-*d*_6_ (100 MHz) | 20 |
| **Figure S40:**^1^H NMR Spectrum of **18** in DMSO-*d*_6_ (400 MHz) | 20 |
| **Figure S41:** ^13^C NMR Spectrum of **18** in DMSO-*d*_6_ (100 MHz) | 21 |
| **Figure S40:**^1^H NMR Spectrum of **19** in DMSO-*d*_6_ (400 MHz) | 21 |
| **Figure S41:** ^13^C NMR Spectrum of **19** in DMSO-*d*_6_ (100 MHz) | 22 |
| **Figure S40:**^1^H NMR Spectrum of **20** in DMSO-*d*_6_ (400 MHz) | 22 |
| **Figure S41:** ^13^C NMR Spectrum of **20** in DMSO-*d*_6_ (100 MHz) | 23 |
| **Figure S40:**^1^H NMR Spectrum of **21** in DMSO-*d*_6_ (400 MHz) | 23 |
| **Figure S41:** ^13^C NMR Spectrum of **21** in DMSO-*d*_6_ (100 MHz) | 24 |

**Figure S1:**^1^H NMR Spectrum of **1** in DMSO-*d*_6_ (400 MHz)

**Figure S2:** ^13^C NMR Spectrum of **1** in DMSO-*d*_6_ (100 MHz)

**
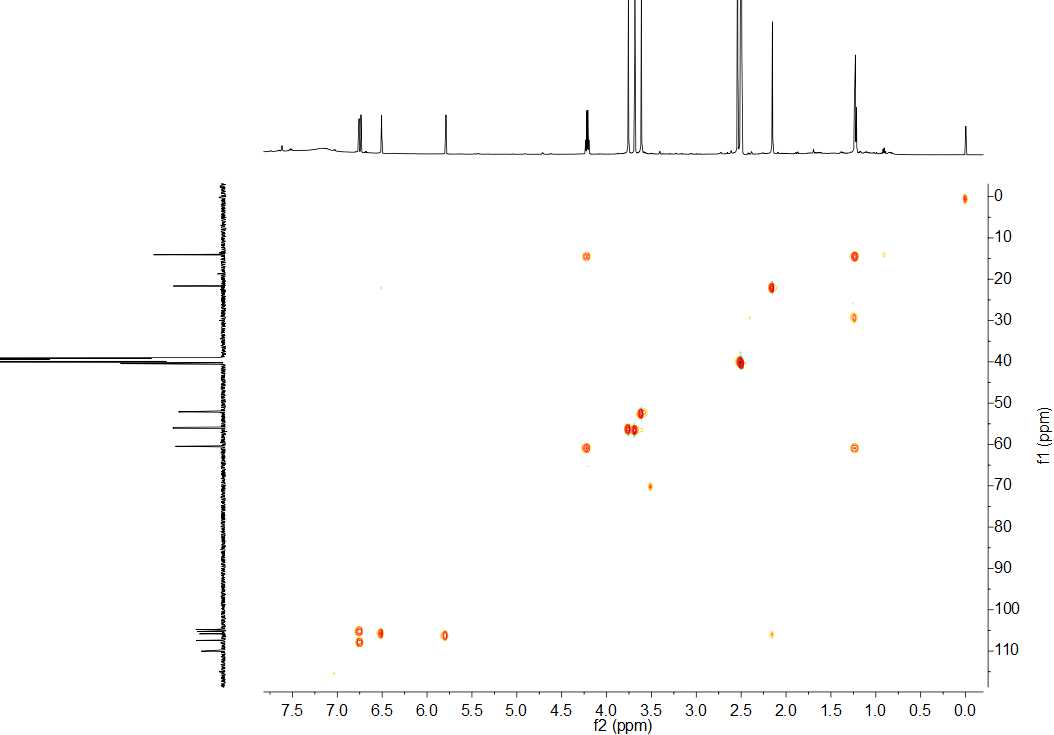
**

**Figure S3**: HSQC Spectrum of **1** in DMSO-*d*_6_

_
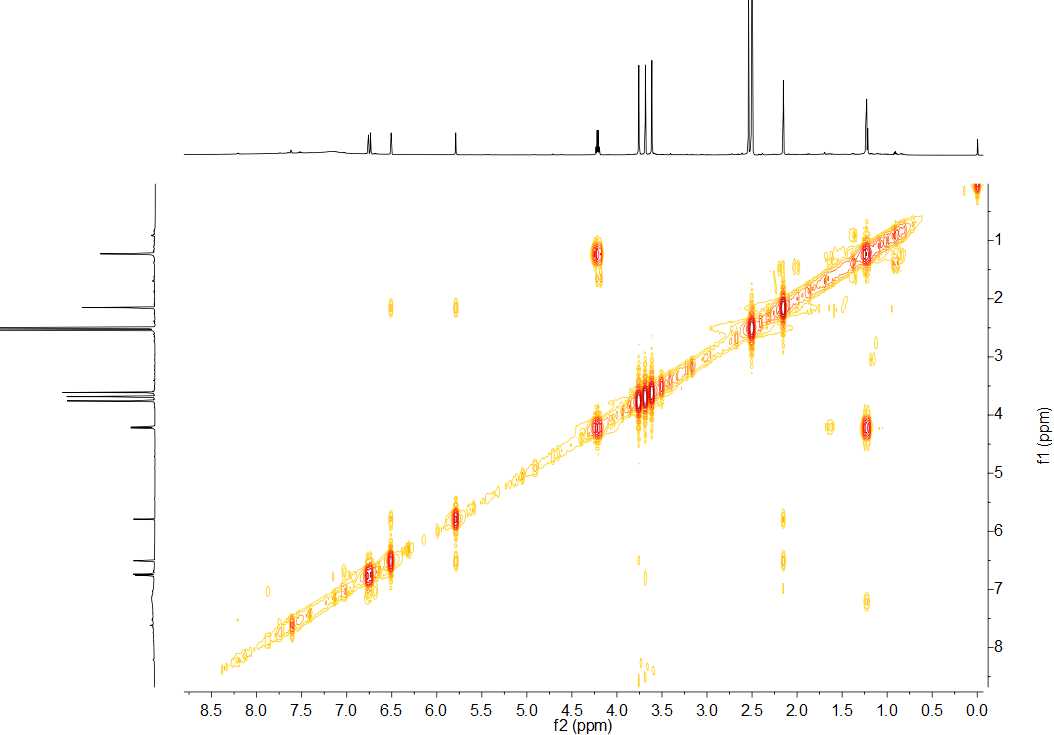
_

**Figure S4:** COSY Spectrum of **1** in DMSO-*d*_6_

**Figure S5**: HMBC Spectrum of **1** in DMSO-*d*_6_

**
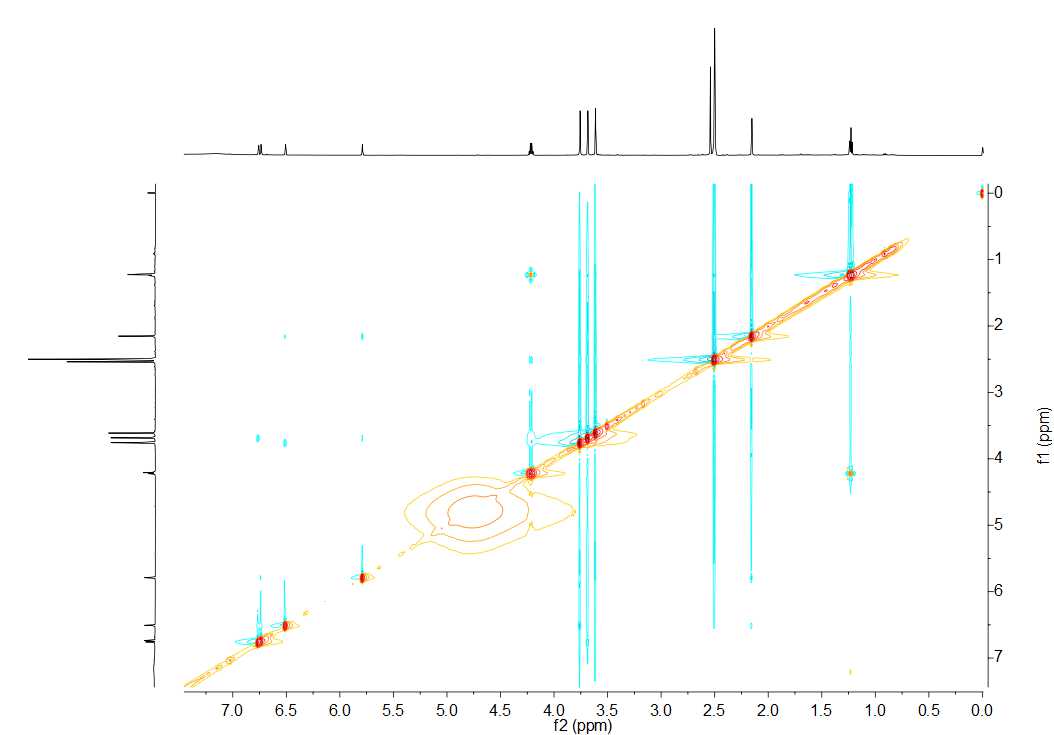
**

**Figure S6**: NOESY Spectrum of **1** in DMSO-*d*_6_


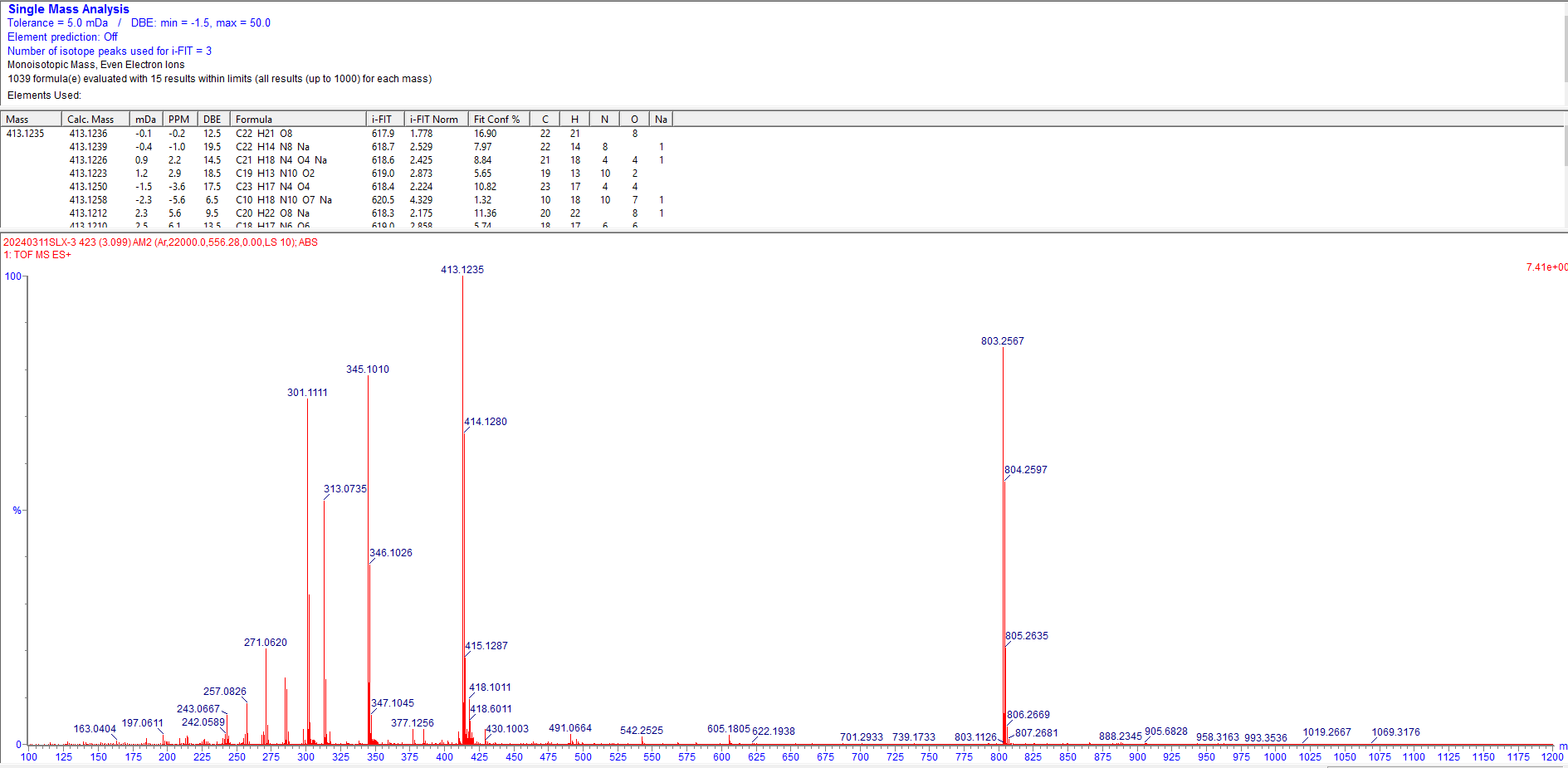


**Figure S7**: HRESIMS Spectrum of **1**

**Figure S8:**^1^H NMR Spectrum of **2** in DMSO-*d*_6_ (400 MHz)

**Figure S9:**^13^C NMR Spectrum of **2** in DMSO-*d*_6_ (100 MHz)

**Figure S10:**^1^H NMR Spectrum of **3** in DMSO-*d*_6_ (400 MHz)

**Figure S11:** ^13^C NMR Spectrum of **3** in DMSO-*d*_6_ (100 MHz)

**Figure S12:**^1^H NMR Spectrum of **4** in DMSO-*d*_6_ (400 MHz)

**Figure S13:** ^13^C NMR Spectrum of **4** in DMSO-*d*_6_ (100 MHz)

**Figure S14:**^1^H NMR Spectrum of **5** in DMSO-*d*_6_ (400 MHz)

**Figure S15:** ^13^C NMR Spectrum of **5** in DMSO-*d*_6_ (100 MHz)

**Figure S16:**^1^H NMR Spectrum of **6** in DMSO-*d*_6_ (400 MHz)

**Figure S17:** ^13^C NMR Spectrum of **6** in DMSO-*d*_6_ (100 MHz)

**Figure S18:**^1^H NMR Spectrum of **7** in DMSO-*d*_6_ (400 MHz)

**Figure S19:** ^13^C NMR Spectrum of **7** in DMSO-*d*_6_ (100 MHz)

**Figure S20:**^1^H NMR Spectrum of **8** in DMSO-*d*_6_ (400 MHz)

**Figure S21:** ^13^C NMR Spectrum of **8** in DMSO-*d*_6_ (100 MHz)

**Figure S22:**^1^H NMR Spectrum of **9** in DMSO-*d*_6_ (400 MHz)

**Figure S23:** ^13^C NMR Spectrum of **9** in DMSO-*d*_6_ (100 MHz)

**Figure S24:**^1^H NMR Spectrum of **10** in DMSO-*d*_6_ (400 MHz)

**Figure S25:** ^13^C NMR Spectrum of **10** in DMSO-*d*_6_ (100 MHz)

**Figure S26:**^1^H NMR Spectrum of **11** in DMSO-*d*_6_ (400 MHz)

**Figure S27:** ^13^C NMR Spectrum of **11** in DMSO-*d*_6_ (100 MHz)

**Figure S28:**^1^H NMR Spectrum of **12** in DMSO-*d*_6_ (400 MHz)

**Figure S29:** ^13^C NMR Spectrum of **12** in DMSO-*d*_6_ (100 MHz)

**Figure S30:**^1^H NMR Spectrum of **13** in DMSO-*d*_6_ (400 MHz)

**Figure S31:** ^13^C NMR Spectrum of **13** in DMSO-*d*_6_ (100 MHz)

**Figure S32:**^1^H NMR Spectrum of **14** in DMSO-*d*_6_ (400 MHz)

**Figure S33:** ^13^C NMR Spectrum of **14** in DMSO-*d*_6_ (100 MHz)

**Figure S34:**^1^H NMR Spectrum of **15** in DMSO-*d*_6_ (400 MHz)

**Figure S35:** ^13^C NMR Spectrum of **15** in DMSO-*d*_6_ (100 MHz)

**Figure S36:**^1^H NMR Spectrum of **16** in DMSO-*d*_6_ (400 MHz)

**Figure S37:** ^13^C NMR Spectrum of **16** in DMSO-*d*_6_ (100 MHz)

**Figure S38:**^1^H NMR Spectrum of **17** in DMSO-*d*_6_ (400 MHz)

**Figure S39:** ^13^C NMR Spectrum of **17** in DMSO-*d*_6_ (100 MHz)

**Figure S40:**^1^H NMR Spectrum of **18** in DMSO-*d*_6_ (400 MHz)

**Figure S41:** ^13^C NMR Spectrum of **18** in DMSO-*d*_6_ (100 MHz)

**Figure S40:**^1^H NMR Spectrum of **19** in DMSO-*d*_6_ (400 MHz)

**Figure S41:** ^13^C NMR Spectrum of **19** in DMSO-*d*_6_ (100 MHz)

**Figure S40:**^1^H NMR Spectrum of **20** in DMSO-*d*_6_ (400 MHz)

**Figure S41:** ^13^C NMR Spectrum of **20** in DMSO-*d*_6_ (100 MHz)

**Figure S40:**^1^H NMR Spectrum of **21** in DMSO-*d*_6_ (400 MHz)

**Figure S41:** ^13^C NMR Spectrum of **21** in DMSO-*d*_6_ (100 MHz)
